# Supplementary material for: Perception and willingness to accept COVID-19 Vaccines: A cross-sectional survey of the general population of Sokoto State, Nigeria
Source: PLoS One. 2022 Dec 1;17(12):e0278332. doi: 10.1371/journal.pone.0278332 (PMC9714919; doi:10.1371/journal.pone.0278332)
Supplement: Attachment — Submitted filename: Response to Reviewers 1.docx [file pone.0278332.s002.docx]

**05/07/2022**

**RESPONSE TO REVIEWERS’ COMMENTS**

**SYNOPSIS OF CORRECTIONS ON “PERCEPTION AND WILLINGNESS TO ACCEPT COVID-19 VACCINES: A CROSS-SECTIONAL SURVEY OF THE GENERAL POPULATION OF SOKOTO STATE, NIGERIA”**

| **Manuscript section** | **Summary of comment by Assessors** | **Actions taken by the candidate/ Explanations** |
| --- | --- | --- |
| Data availability | If there are ethical or legal restrictions on sharing a de-identified data set, please explain them in detail | There are no ethical or legal restrictions on the data from the study |
| ORCID id | PLOS requires an ORCID iD for the corresponding author in Editorial Manager on papers submitted after December 6th, 2016. Please ensure that you have an ORCID iD | The ORCID iD was included after the title of the paper as Orcid:0000000223396998 |
| ABSTRACT | Why were respondents less than 18years included in the study?  The abstract is unstructured | Actually the least age of the respondents was 18 years which can vote and be married  The abstract has been structured |
| INTRODUCTION | - The author mentioned the prevalence of COVID 19 as at 10th of February This is an old statistics - The introduction is bereft of justification to the study | - This has been updated as at 27^th^ June, 2022 - Justification of the study has been included in the tail end of the introduction |
| METHODS | - There is no indication of the number of variables used to assess perception of covid-19 vaccine - How did the authors calculate perception and hesitancy - Explain also how willingness to receive COVID-19 vaccine was derived - Include sample size, (n=883) under Frequency - Authors are advised to include logistic regression analysis in the result. - An explanation may also be required on how the sampling interval for the systematic random sampling technique was derived - How were the participants selected - Include the ethical approval number from the Health Research and Ethics Committee - What information was provided to the respondents on participation in the study, confidentiality, benefits and risks associated with   participation in the study?   - There was gender bias in the study - Use the Chi square sign instead of Test statistics - Explain others under ethnicity and religion as footnotes - Reduce the age groupings to three or four (Three will be more meaningful) - The Table on factors affecting perception should come first before that for willingness | - This has been captured in the methods section as eight variables were used and the method of assessment outlined - These were explicitly explained in tail end of the methods section - Willingness was assessed based on the question “if the respondents would like to be vaccinated” in which 636(72.4) agreed that they would like to be vaccinated - This was effected - This has been include as Table 6 - This has been explained in the methods section for sampling frame and interval - The participants were selected using both the modified Lots Quality Assurance Survey and systematic sampling methods - Ethical approval number has been included in the ethical approval section (SKHREC/021/2021) - This has been included at the end of the methods section under Ethical approval - Tried as much as possible to have equal representation of both sexes, it was difficult as some women would not accept to be interviewed in the absence their husbands while some were skeptical about the purpose of the interview despite explanations on the purpose of the study - This has been effected - These have been explained as footnotes - These have been effected in the relevant tables - This has been effected |
| DISCUSSION | - “This herd immunity can be achieved through a threshold range of between 50-65% [61,62].” The cited literatures did not consider the emergence of COVID variants. Please refer to the recent literatures such as this one: Caldwell et al. Paediatric Respiratory Reviews2021; 39: 32-39 – doi: 0.1016/j.prrv.2021.07.002 - Findings that only 9.2% believed COVID-19 was man-made. Then, authors compared the finding with the fact that 67% respondents of the previous study felt the exaggeration of COVID-19 threat. The two findings are NOT relevant, therefore not comparable. This is a major weakness of the manuscript. I strongly suggest author to make revision of the entire discussion regarding this matter. - A study from Rosiello et al. (Narra J 2021; 1(3): e55-doi: 10.52225/narra.v1i3.55) suggests that population of North African region are still low in vaccine acceptance. Please incorporate this study in your discussion | - The discussion has been modified to include the issue of Variants of Concern(VoC) as posited by Caldwell et al - This has been revised with a new literature included for the comparison - This has been incorporated as reference 56 |
| CONCLUSION | The conclusion was verbose and there was no recommendation | The conclusion and recommendations have been modified |
