# Supplementary material for: Perception and willingness to accept COVID-19 Vaccines: A cross-sectional survey of the general population of Sokoto State, Nigeria
Source: PLoS One. 2022 Dec 1;17(12):e0278332. doi: 10.1371/journal.pone.0278332 (PMC9714919; doi:10.1371/journal.pone.0278332)
Supplement: Attachment — Submitted filename: Review comments.docx [file pone.0278332.s003.docx]

**Review comments**

**General comment**

Authors are commended for the good review of the manuscript. However, there are still some work to be done before the manuscript could be accepted for publication.

Authors should critically review the manuscript for some errors of grammar and pay attention to the following points:

**Abstract**

**Results**

Be consistent with number of decimal places all through the manuscript. Check the result section also.

In the methods section of the abstract explain how perception of COVID-19 was assessed

Instead of poor perception, report good perception

Instead of up to 72.4%, indicate that majority of the respondents……

Include Nigeria as a key word

**Introduction**

Be mindful of use of capital letters, note Pandemic in first line of introduction section

**Materials and methods**

Be consistent on use of capital letters for the senatorial zones

Let us have the projected population of Sokoto state for 2022

**Sampling technique**

Under sample size, the Authors indicated cluster sampling technique was used in the study and applied a design effect of 2.5.However under sampling method, Authors indicated that a systematic sampling technique was used to select the respondents. These two aspects of the manuscript should be reconciled since a probability sampling technique was used to select the respondents.

Use of capital ‘P’ for participants should be reviewed

**Instrument and method of data collection**

Use questionnaire instead of questionnaires in second line

Eight variables were used to assess perception of COVID-19 vaccine among the respondents. How could respondents have a score of three or below? This should be explained. Authors should explain how good or poor perception was derived.

Review the use of mean and standard deviation in the context they were presented in this sub-section.

**Results**

Use No formal education instead of None

Explain ‘Others’ as footnote

**Figure 3**

Why was 0.352 used beside the bar and 35.2% used in the reporting? This could be confusing.

**Table 4**

Use variable instead of variables

Good perception should be in the first column instead of poor perception

Include sample size (n=..) under perception of COVID-19 vaccine

Use three decimal places for Chi square all through the manuscript

‘Unmarried’ is a derived variable and should be explained as a footnote

Use No formal education

Could ‘Others’ under ethnicity be ethnic minorities?

Apply these changes for Table 5 also

In table 5, Good perception should be the first category instead of poor perception

Why was Residence (Urban/Rural) not included as an independent variable in tables 4 and 5?

**Table 6**

Authors should state the criterion for inclusion of variables into the logistic regression model. I advise on the use of p values <0.2 on bivariate analysis, (Chi square test) Why was perception of COVID-19 vaccine not included in this model.

Table 6 should be reviewed in this context. Since logistic regression analysis has been included in this study, Authors should include this result in the abstract by reporting adjusted odds ratios instead of p values.

For table 6, use adjusted odds ratio instead of adds ratio.

Authors could perform logistic regression analysis for perception of COVID-19 vaccine but the focus of this study should be that for willingness. I advise Authors to combine the table for bivariate and multivariate logistic regression analysis for each of the outcome variables so as to minimize the number the tables.

**Discussion**

Authors should organize this section into distinct paragraphs

The revisions above should be reflected in the discussion.

**Conclusion**

Focus should be on reporting ‘positives’

Review the use of a ‘handful’
