# Supplementary material for: Perception and willingness to accept COVID-19 Vaccines: A cross-sectional survey of the general population of Sokoto State, Nigeria
Source: PLoS One. 2022 Dec 1;17(12):e0278332. doi: 10.1371/journal.pone.0278332 (PMC9714919; doi:10.1371/journal.pone.0278332)
Supplement: Attachment — Submitted filename: Response to Reviewer query.docx [file pone.0278332.s004.docx]

**AUTHORS’ RESPONSE TO REVIEWER’S QUERIES/OBSERVATIONS**

| **Reviewer’s comment** | **Authors’ response** |
| --- | --- |
| Include sample size (n=) under perception | Sample size (n=) already included |
| Use three decimal places for Chi square all through the manuscript | Corrected to 3 decimal places |
| ‘Unmarried’ is a derived variable and should be explained as a footnote | Unmarried” explained as footnote |
| Use No formal education | No formal education used instead of non-formal education |
| Could “others” under ethnicity be ethnic minorities? | Others under ethnicity implied ethnic minorities which are too numerous to mention |
| Apply these changes in table 4 to Table 5 also | Done |
| In table 5, Good perception should be the first category instead of poor perception | Done |
| Why was Residence (Urban/Rural) not included as an independent variable in tables 4 and 5? | Residence included in tables 4 and 5 |
| Authors should state the criterion for inclusion of variables into the logistic regression model. I advise on the use of p values <0.2 on bivariate analysis, (Chi square test) Why was perception of COVID-19 vaccine not included in this model | Criterion stated  Perception is included in the model |
| Since logistic regression analysis has been included in this study, Authors should include this result in the abstract by reporting adjusted odds ratios instead of p values. | Done |
| For table 6, use adjusted odds ratio instead of adds ratio. | Authors used adjusted odds ratio but did not indicate it in the table; that has been done now |
| Authors could perform logistic regression analysis for perception of COVID-19 vaccine but the focus of this study should be that for willingness. I advise Authors to combine the table for bivariate and multivariate logistic regression analysis for each of the outcome variables so as to minimize the number the tables | Authors have done the analysis for predictors of perception also, however, they agreed to leave it out due to the number of tables and merging the tables will make the table complex and longer; moreover the main focus of the work is willingness rather than perception |
